# The Role of Bone and Root Resorption on the Biomechanical Behavior of Mandibular Anterior Teeth Subjected to Orthodontic Forces: A Finite Element Approach

**DOI:** 10.3390/biomedicines12091959

**Published:** 2024-08-28

**Authors:** Jana Flatten, Thomasz Gedrange, Christoph Bourauel, Ludger Keilig, Anna Konermann

**Affiliations:** 1 Oral Technology, University Hospital Bonn, 53111 Bonn, Germany; 2 Department of Orthodontics, University Hospital Dresden, 01307 Dresden, Germany; 3 Department of Prosthetic Dentistry, Preclinical Education and Materials Science, University Hospital Bonn, 53127 Bonn, Germany; 4 Department of Orthodontics, University Hospital Bonn, 53111 Bonn, Germany

**Keywords:** bone resorption, orthodontics, periodontitis, root resorption, side effects, tooth movement

## Abstract

Aims: This study was conducted to systematically evaluate the biomechanical impact of varying degrees of root and bone resorption resulting from periodontitis and orthodontic tooth movement (OTM) on the mandibular anterior teeth. The objective was to determine whether these distinct resorption patterns exert a specific influence on tooth displacement and strain patterns. Methods: A finite element (FE) model of an idealized anterior mandible from the first premolar in the third to the fourth quadrant was developed without bone or root resorption and a constant periodontal ligament (PDL) thickness of 0.2 mm. Variations included three root resorption levels (0%, 20%, 50%) and three bone resorption types (circular 50%, circular 80%, vestibular 80%). Models ranged from 200,000 to 440,000 elements and 55,000 to 130,000 nodes. Orthodontic forces, namely root torque (5 Nmm), intrusion (0.2 N), and distalization (0.5 N) were applied for subsequent crown displacement and PDL strain analysis. Results: A total of 180 simulations were performed. Simulations showed that displacement was similar across different bone resorption conditions, irrespective of modeled root resorptions. Circumferential bone resorption increased tooth displacement, regardless of root resorption status. Vestibular bone resorption exhibited less increase in tooth displacement. However, when accompanied by root resorption, the combination exacerbated tooth displacement. Strains in the PDL clearly increased with a circumferential bone resorption of 80%. Conclusions: This study highlights the critical role of bone resorption in tooth displacement during OTM, particularly the challenges associated with circumferential resorption. Clinicians must consider both bone and root resorption for personalized medicine treatment of patients with severe periodontitis, in favor of low-force application strategies to optimize outcomes and minimize complications linked to excessive tooth displacement.

## 1. Introduction

Orthodontic tooth movement (OTM) involves a biomechanical process where the application of orthodontic forces effect the displacement of teeth within the alveolar bone driven by an accelerated remodeling of the periodontal ligament (PDL) and bone, which is orchestrated by cellular and molecular responses to the applied mechanical stimuli [1]. Mechanical loading of a tooth induces force transmission to the PDL, the interface between tooth and bone, resulting in micro-injuries to this soft tissue and the subsequent initiation of an acute sterile inflammatory response [1,2]. Both tensile and compressive forces are exerted, eliciting coordinated bone resorption on the compression side and bone formation on the tension side [3].

Excessive compressive forces generated by orthodontic treatment can contribute to heightened osteoclastogenesis and thus lead to pathological tissue resorption [4]. This side effect, termed external root resorption (ERR), commences with the resorption of the superficial root cementum and may progress to encompass the underlying dentin in more severe stages, marking an irreversible stage once dentin is affected. The scoring index for root resorption comprises four stages [5]. Stage 1 is marked by irregular root contour, Stage 2 involves apical root resorption less than 2 mm of the original root length, Stage 3 indicates apical root resorption ranging from 2 mm to 1/3 of the original root length, and Stage 4 signifies apical root resorption exceeding 1/3 of the original root length. However, investigations have shown that a significant number of teeth exhibit irregularities in the apical root contour, even in the absence of orthodontic treatment, prompting emphasis on Stages 2 to 4 for categorizing ERR [6]. ERR undergoes repair by cellular cementum, however, it can result in the permanent diminution of root length [7]. Investigations indicate that as many as 90% of teeth undergoing orthodontic treatment experience varying degrees of apical external root resorption, with up to 10% of cases exhibiting severe apical resorption exceeding 4 mm [8]. Given the substantial reduction in the area of periodontal attachment per millimeter of root length from the cervical to the apical region of a tapering root, the loss of periodontal attachment due to 3 mm of apical external root resorption is assessed to correspond to approximately 1 mm of cervical bone loss [9].

In addition to OTM, additional systemic and local risk factors contributing to the onset of ERR exist, with chronic periodontitis demonstrating a particularly clear correlation [7,10,11]. Notably, root resorption occurs three times more frequently in teeth affected by periodontal diseases compared to those unaffected [10]. Chronic periodontitis is a highly prevalent and progressive form of periodontal disease characterized by chronic inflammation of the gingiva and surrounding tissues, leading to gradual and irreversible destruction of the periodontium including alveolar bone resorption and gingival attachment loss to the tooth surface [2]. Investigations unveiled that 60% of teeth diagnosed with varying severities of chronic periodontitis displayed ERR, particularly affecting mandibular incisors. The occurrence and extent of this resorption phenomenon correlated directly with the extent of periodontal tissue destruction [10]. Periodontitis leads to the loss of alveolar bone and attachment of the periodontium, resulting in notable tooth displacement. Teeth with increased displacement are significantly more susceptible to developing ERR due to heightened mechanical irritation of the root surface, particularly in the exposed apical third. This phenomenon is caused by a lack in periodontal fibers and alveolar bone for support, reducing the ability of the teeth to withstand occlusal forces and facilitating the onset of resorption [10].

Nevertheless, the presumption of a direct and linear correlation between the magnitude of bone resorption and the extent of ERR along with the resulting tooth displacements and strains during OTM across various movement scenarios constitutes a research question that remains to be systematically and comprehensively elucidated.

Understanding the underlying dynamics and biomechanical effects of resorption is crucial for developing more effective orthodontic treatments and interventions aimed at mitigating the adverse effects of these processes. In this investigation, finite element (FE) models were utilized to assess the concurrent impact of varying degrees of chronic periodontitis-induced bone resorption and ERR during simulated OTM on resultant forces, as much as their influence on the biomechanical behavior of the anatomical structures tooth, PDL and alveolar bone. The scope of this study was to systematically investigate the influence of different degrees of combined root and bone resorption on the biomechanical behavior of teeth in the lower jaw in an idealized numerical model, especially during orthodontic treatment.

## 2. Material and Methods

**Figure 1:**
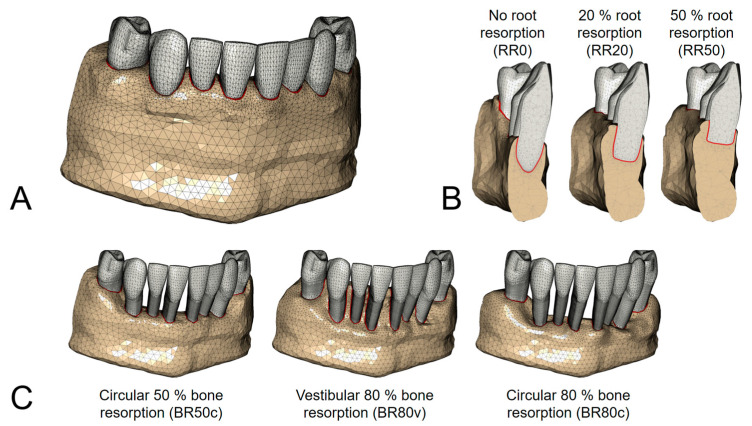
Initial simulated model of the anterior mandible starting from the first premolars without any bone or root resorption (**A**). Modeling of three degrees of root resorption: no resorption (RR0), 20% resorption (RR20), and 50% resorption (RR50) (**B**). Modeling of three degrees of bone resorption relative to the remaining root length: circular bone resorption of 50% (BR50c), circular bone resorption of 80% (BR80c), and vestibular bone resorption of 80% (BR80v) (**C**).

The generated FE model was based on a commercially available surface dataset of a lower jaw (“teeth with roots and gum”, Viewpoint Data Labs [now Digimation Inc., Lake Mary, FL, USA]). The initial simulated model generated for this study represented an idealized structure of the anterior mandible, spanning from the first premolar in the third quadrant to the first premolar in the fourth quadrant, which is presented in Figure 1A. This model served as a baseline without any bone or root resorption. As the PDL was not present in the original model, an idealized PDL was generated with a constant thickness of 0.2 mm. Following this, three degrees of root resorption were modeled in accordance with the literature including no resorption (RR0), 20% resorption (RR20), and 50% resorption (RR50) [12,13]. Subsequently, three patterns of bone resorption, corresponding to the residual root length, were simulated in alignment with prior research categorizing moderate generalized bone loss (26–50%) and severe generalized bone loss (>50%) [14]. Thus, models comprised a circular bone resorption of 50% (BR50c), circular bone resorption of 80% (BR80c), and vestibular bone resorption of 80% (BR80v). These specific configurations are illustrated in Figure 1B,C.

Depending on the modeled bone resorption, the resultant models comprised between 200,000 and 440,000 elements and 55,000 to 130,000 nodes. Specifically, the bone was represented by 70,000 to 100,000 elements and 15,000 to 30,000 nodes, teeth were modeled using 90,000 to 180,000 elements and 25,000 to 50,000 nodes, and the PDL was defined by 50,000 to 150,000 elements and 15,000 to 50,000 nodes. Four-noded tetrahedral elements were employed for the bone and teeth, while the PDL was represented using degenerated eight-noded hexahedral elements. Both bone and tooth materials were considered homogeneous and isotropic. The material behavior of the PDL was characterized using a previously established bilinear elastic model [15,16,17,18]. Details of the material parameters used in all simulations are provided in Table 1.

**Figure 2:**
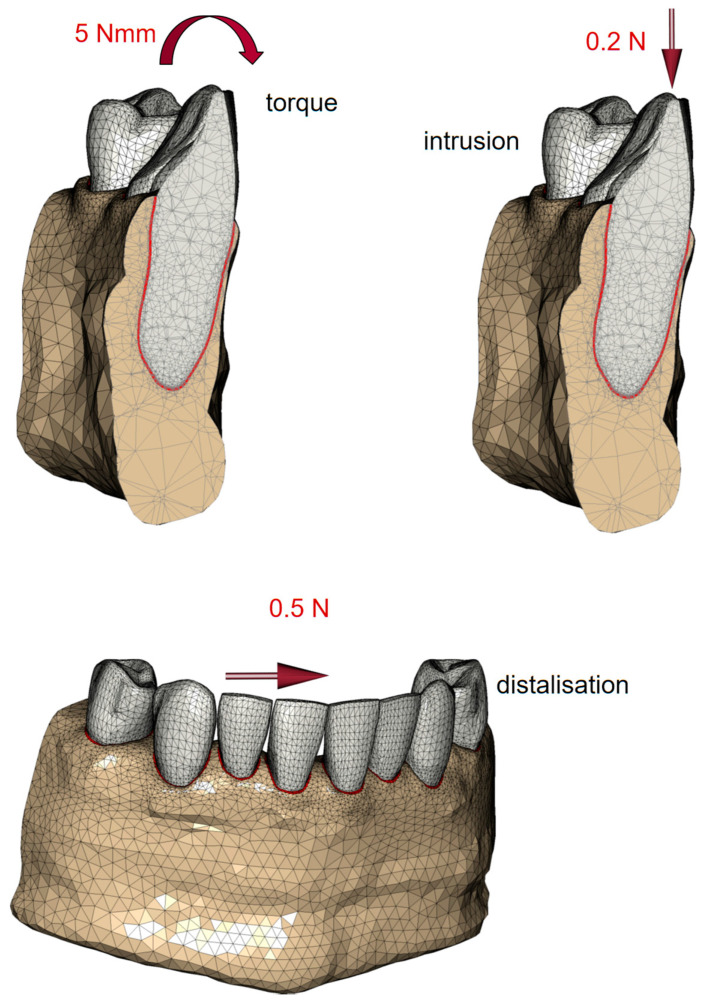
Simulated loading of each incisor and canine with orthodontic forces: root torque with 5 Nmm, intrusion with 0.2 N, and distalization with 0.5 N.

Simulated loading of each incisor and canine with therapeutically validated orthodontic forces was performed separately to realize three different initial movement directions, namely the root torque with 5 Nmm, intrusion with 0.2 N, and distalization with 0.5 N, as depicted in Figure 2 [19,20,21,22]. In the subsequent analyses, crown displacement as much as the strains in the PDL were determined. During all simulations, potential touching contact (Coulomb stick-slip) was permitted between adjacent crowns to simulate possible interactions, particularly under the influence of distalization forces. The resulting crown movements and the equivalent strains in the PDL were recorded throughout the simulations. The finite element analysis was conducted using the MSC Marc/Mentat version 2020 software package (Hexagon AB, Stockholm, Sweden).

## 3. Results

**Figure 3:**
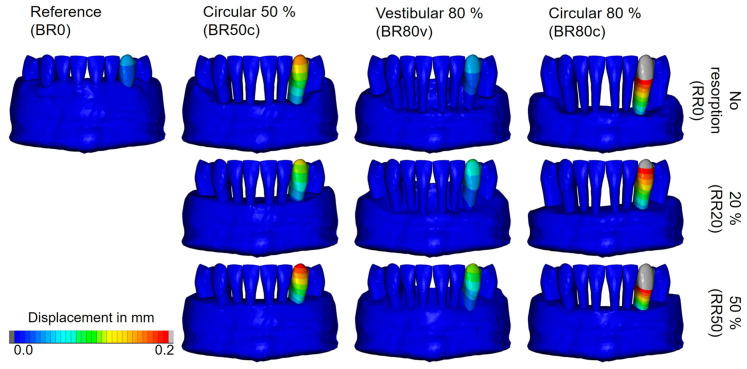
Calculated displacements of the lower left canine under a torque moment of 5 Nmm across models with varying degrees of bone resorption (columns) and root resorption (rows). No root resorption (RR0), 20% root resorption (RR20), and 50% root resorption (RR50). No bone resorption (BR0), circular bone resorption of 50% (BR50c), circular bone resorption of 80% (BR80c), and vestibular bone resorption of 80% (BR80v).

A total of 180 simulations were conducted to determine the initial tooth movement in response to the specified orthodontic forces. To exemplify the overall patterns observed in the simulations, Figure 3 presents the displacement of the lower left canine as a representative case. This figure depicts the computed displacements under an applied torque moment of 5 Nmm across models characterized by different levels of bone and root resorption. The different bone resorption scenarios represented in the columns exhibit similar displacement magnitudes, independent of the modeled root resorption. In contrast to the research assumption, this observation indicates that bone resorption may not linearly correlate with the extent of tooth displacement across varying models, as the magnitude of displacement remained relatively consistent.

**Figure 4:**
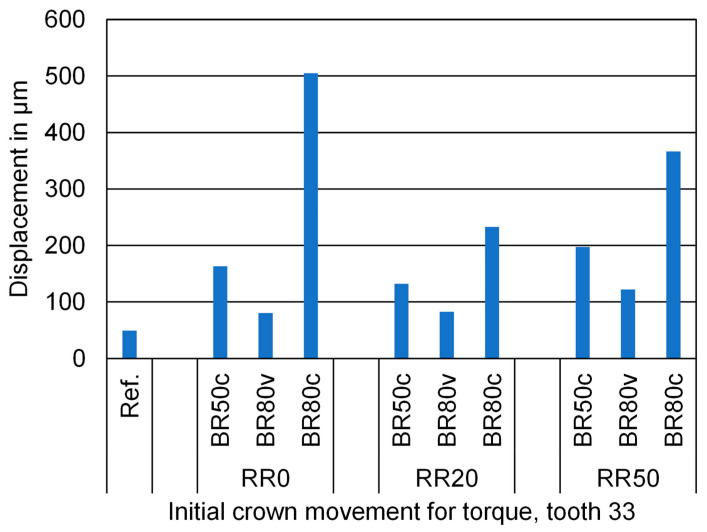
Numerical values of the initial crown displacement for the lower left canine under a torque moment of 5 Nmm indicated on the *y*-axis in micrometer (μm). No root resorption (RR0), 20% root resorption (RR20), and 50% root resorption (RR50). No bone resorption (Ref.), circular bone resorption of 50% (BR50c), circular bone resorption of 80% (BR80c), and vestibular bone resorption of 80% (BR80v).

Conversely, the root resorption scenarios represented in the rows did not reveal a distinct pattern. The numerical values of the initial crown displacement for these simulations are provided in Figure 4. Detailed analysis revealed that circumferential bone resorption substantially increased tooth displacement, regardless of the presence or absence of root resorption, and independent of the extent of any existing root resorption. This finding partially aligns with the expected correlations, revealing a significant influence of circumferential bone resorption on tooth displacement. However, it also underscores that root resorption does not consistently affect tooth displacement across all conditions. Conversely, vestibular bone resorption resulted in a less pronounced increase in tooth displacement. However, when vestibular bone resorption was accompanied by root resorption, there was a more significant increase in mobility, indicating a synergistic effect. This suggests that the combined presence of vestibular bone loss and root resorption amplifies tooth displacement more than either condition in isolation, with the extent of root resorption being a critical factor in determining overall displacement when vestibular bone resorption is also present. This finding further addresses the research question by indicating that, while the interaction between vestibular bone resorption and root resorption can significantly influence tooth displacement, the effect is complex and contingent on the specific interplay of these conditions.

**Figure 5:**
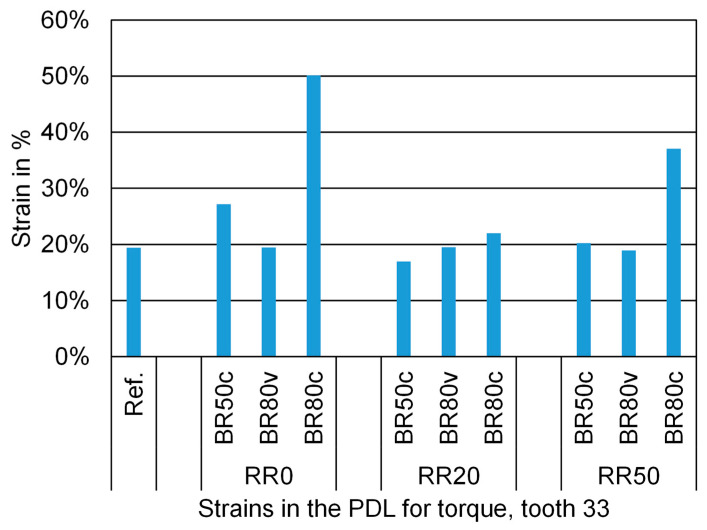
Numerical values of the initial strains in the PDL of the lower left canine under a torque moment of 5 Nmm indicated on the *y*-axis in percent (%). No root resorption (RR0), 20% root resorption (RR20), and 50% root resorption (RR50). No bone resorption (Ref.), circular bone resorption of 50% (BR50c), circular bone resorption of 80% (BR80c), and vestibular bone resorption of 80% (BR80v).

Investigations revealed that the increase in mechanical strains within the PDL and surrounding structures did not correspond to the extent of tooth displacement. Figure 5 illustrates the resulting strains in the PDL of the loaded canine in the same simulations. Notably, the differences in strains between the reference model and the models with bone and/or root resorption were less pronounced compared to the observed displacements. This discrepancy suggests that while tooth displacement increases due to bone resorption, the internal stresses and strains within the supporting structures do not increase proportionally, thus addressing the research question by showing a lack of direct correlation between the strains and displacement. However, the strains in the PDL markedly increased with 80% circumferential bone resorption, indicating that, under conditions of severe bone loss, there is likely a stronger correlation between bone resorption, heightened strains, and tooth movement. This finding provides valuable insights into the complexity of the research question, highlighting how severe bone resorption can significantly impact the relationship between these variables.

**Figure 6:**
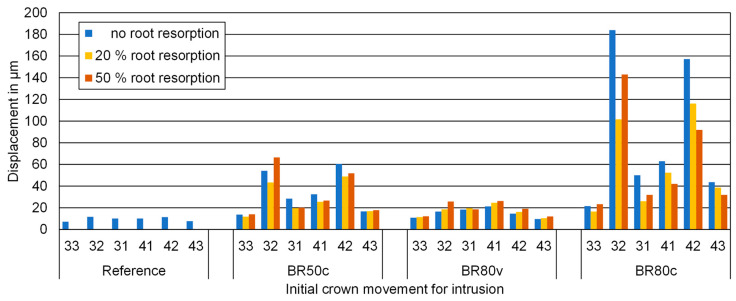
Numerical values of the initial crown displacements during intrusion for all teeth and models investigated indicated on the *y*-axis in micrometer (μm). No root resorption (RR0), 20% root resorption (RR20), and 50% root resorption (RR50). No bone resorption (Reference), circular bone resorption of 50% (BR50c), circular bone resorption of 80% (BR80c), and vestibular bone resorption of 80% (BR80v).

Crown displacements during intrusion for all teeth and models, as displayed in Figure 6, were markedly impacted by circular bone resorption compared to bone resorption confined to the vestibular side. Crown displacement noticeably increased with circumferential bone resorption, both at the 50% and 80% levels of bone loss, indicating a direct correlation between the extent of bone resorption and increased tooth displacement during orthodontic intrusion, with more severe resorption leading to greater displacement. This observation supports the assumption posited in this study by demonstrating a clear relationship between the magnitude of bone resorption and tooth displacement, at least in the context of circumferential bone loss.

The displacement varied among the types of loaded teeth, with incisors exhibiting greater movement than canines. Notably, the second incisor showed the most pronounced crown movement. This behavior was generally symmetrical between the teeth on the left and right sides. While these findings reinforce the notion that bone resorption correlates with increased displacement, they also suggest that the type of tooth plays a significant role. Although different grades of root resorption influenced displacement, they did not lead to a systematic increase or decrease in movement, suggesting that while root resorption does contribute to tooth displacement, its effects are more variable and less predictable compared to those of bone resorption. This variability indicates that the presumption of a direct and linear correlation between root resorption and tooth displacement is not fully supported, highlighting that the study question remains only partially answered in this regard.

**Figure 7:**
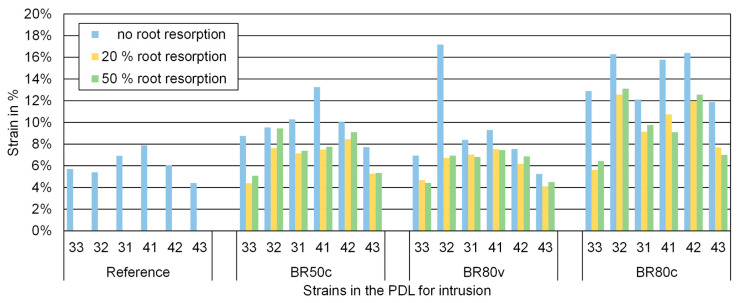
Numerical values of the initial strains in the PDL during intrusion for all teeth and models investigated indicated on the *y*-axis in percent (%). No root resorption (RR0), 20% root resorption (RR20), and 50% root resorption (RR50). No bone resorption (Reference), circular bone resorption of 50% (BR50c), circular bone resorption of 80% (BR80c), and vestibular bone resorption of 80% (BR80v).

The strains in the PDL for the same simulations are shown in Figure 7. As observed with the torque simulations, strains increased in models with bone resorption compared to the reference model, though this increase was less pronounced than the initial crown movement. This finding suggests that while bone resorption does correlate with increased strains, the relationship is not as pronounced or linear as the correlation observed with tooth displacement.

In most simulations with root resorption, the calculated strains were lower than in the corresponding models with the same bone resorption rate but without root resorption, indicating that root resorption does not consistently amplify strains in the PDL, thus challenging the assumption of a straightforward relationship between ERR, strains, and tooth movement.

**Figure 8:**
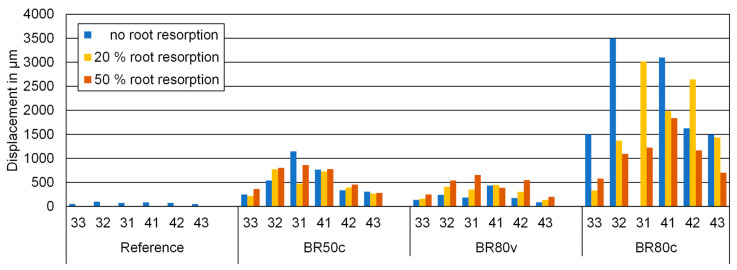
Numerical values of the initial crown displacements during distalization for all teeth and models investigated indicated on the *y*-axis in micrometer (μm). No root resorption (RR0), 20% root resorption (RR20), and 50% root resorption (RR50). No bone resorption (Reference), circular bone resorption of 50% (BR50c), circular bone resorption of 80% (BR80c), and vestibular bone resorption of 80% (BR80v).

The initial crown movement subsequent to applying a distalization force demonstrates that, consistent with previous findings, the canines exhibited markedly lower mobility compared to the incisors, which is shown in Figure 8. The impact of bone resorption was similar to previous findings, as vestibular bone resorption resulted in the smallest increase in displacement, whereas 80% circumferential bone resorption caused the highest increase in displacement. This consistency across different movement scenarios reinforces the idea that bone resorption, particularly circumferential, plays a dominant role in determining tooth displacement, thereby partially fulfilling the research question by establishing a more predictable relationship between bone resorption and displacement. However, the non-linear and variable effects of root resorption indicates that further research is needed to comprehensively elucidate the full dynamics involved.

## 4. Discussion

Impact of ERR and chronic periodontitis on the altered biomechanical behavior of teeth

The results of this study underscore the predominant influence of bone resorption on the initial tooth displacement during root torque, intrusion as much as distalization movement, with circumferential resorption having the most severe effects. Increased strains within the periodontal ligament (PDL) resulting from heightened displacement, as observed in several simulations, highlight practical limits to the forces that can be applied to compromised teeth. This underscores the necessity of a balanced approach in orthodontic force application. A thorough understanding of these interactions is essential for clinicians in planning and implementing orthodontic treatments as it emphasizes the complex biomechanical responses of teeth to varying types and extents of resorption and their cumulative effects on both tooth displacement and internal strains. Even though ERR does not exert as pronounced an influence as bone resorption on the altered biomechanical behavior of teeth, it still warrants significant attention due to the frequent co-occurrence of these two phenomena. Research has demonstrated that teeth with severe chronic periodontitis exhibit more transient ERR than those with milder forms of the disease. Over 80% of teeth with severe chronic periodontitis, and consequently significant bone resorption, showed signs of ERR, whereas the severity of periodontitis is a key determinant in the presence and extent of ERR [11]. This underscores the importance of considering both bone resorption and ERR in clinical assessments and treatment planning for periodontal and orthodontic conditions. However, idiopathic causes are also possible, particularly as occlusal as much as orthodontic forces can induce ERR, especially in teeth affected by severe periodontitis [11,23]. Teeth with severe periodontitis naturally experience high occlusal trauma due to their increased mobility, and since resorption often results from traumatic injuries, a higher percentage of these teeth exhibit resorption. Previous studies have consistently found that teeth with chronic periodontitis most frequently show resorption in the apical third [10,24]. This phenomenon can be explained by considering the biomechanical properties and differential stress distributions of the alveolar bone. Specifically, the alveolar bone is comparatively thinner and exhibits greater elasticity in the apical third of the root. Consequently, it is required to absorb a significantly higher amount of the mechanical stress induced by orthodontic forces. In contrast, the alveolar bone surrounding the cervical third of the root is thicker and exhibits less elasticity, thereby subjecting it to a relatively lower mechanical load [7].

Regarding the depth of resorptions, one study found that 47.2% of resorptions in the control teeth reached the dentin compared to 73.6% in orthodontically moved teeth [25]. These findings underscore the complex relationship between the severity of periodontal disease, the application of orthodontic forces, and the extent of ERR. It is crucial for clinicians to carefully consider these factors to minimize the risk of resorption and optimize treatment outcomes.

Limitations of the Study

The present study has several limitations that should be carefully considered. Firstly, the geometric modeling of bone and root resorption was highly specific, focusing on particular shapes that, while representative, do not encompass the full spectrum of resorption patterns observed clinically. In practice, resorption can present in a variety of geometries, and the chosen model may not fully capture the diversity of these presentations. Secondly, this study focused exclusively on analyses of the mandibular front teeth, which limits the generalizability of the results to all teeth. However, this focus is particularly relevant given that previous studies have identified mandibular incisors as the teeth most commonly affected by ERR, as they are single-rooted, possess a slender anatomy, and have fewer surrounding periodontal structures, making them more susceptible to periodontal destruction [10]. This anatomical vulnerability was a key reason for selecting mandibular incisors for this analysis, as their susceptibility to ERR provides important insights into the mechanisms and effects of bone and root resorption in orthodontic contexts. Finally, the use of relative bone height as a metric for assessing resorption introduces challenges in comparing different geometries, and thus represents another restriction of the investigations. This approach may obscure the nuances of how various resorption patterns influence tooth displacement and strain distribution within the PDL, limiting the generalizability of the findings.

Future Research Directions

Addressing these limitations in future research could enhance the applicability and clinical relevance of the findings, contributing to more refined and effective orthodontic treatment protocols. Moreover, future research could explore the direct translation of these findings into clinical practice, particularly through the development of tailored orthodontic interventions that account for each patient’s unique resorption patterns and periodontal health. By investigating how these biomechanical insights can be integrated into personalized treatment plans, researchers may uncover strategies that not only improve treatment outcomes, but also mitigate potential complications such as excessive root resorption or unintended tooth displacement. Additionally, incorporating advanced imaging techniques and computational modeling could allow for the precise assessment of individual patient anatomy, leading to more predictive and adaptive treatment approaches. This line of research holds promise for advancing the field of orthodontics toward a more patient-specific, precision-based practice, ultimately enhancing both the safety and effectiveness of orthodontic care.

Advances in FE Analysis

The examination of stress and strain responses to orthodontic forces using FE analysis is not novel in itself, however, earlier implementations of FE methodology were limited in resolution, comprising only 240 elements for an entire tooth [26]. Significant advancements in the present study distinguish it from these earlier efforts, as most notably, a highly detailed FE model incorporating 90,000 to 180,000 elements and 25,000 to 50,000 nodes was employed. This substantial increase in model complexity enhanced the precision and degree of detail of the stress distribution analysis, allowing for the detection of subtle biomechanical nuances that may be missed by less sophisticated models. Moreover, this investigation is distinguished by its analysis of varying degrees of bone and root resorption, with an emphasis on the simultaneous consideration of both factors. While prior studies have also examined initial tooth movement in relation to different root lengths and alveolar bone heights, they have limited their focus to a single maxillary anterior tooth as a representative model [27,28,29]. In contrast, our study overcame these limitations by analyzing the entire mandibular anterior segment, which is of greater clinical relevance due to the inherently fragile and resorption-prone nature of mandibular anterior roots. Additionally, this research is pioneering in its exploration of the impact of different orthodontic forces on subsequent crown displacement and PDL strain. Special emphasis was placed on the three most critical forces from an orthodontic perspective with respect to root resorption development, namely root torque, intrusion, and distalization, thereby addressing the gaps left by previous studies and providing a more thorough understanding of the biomechanical challenges in orthodontic treatment [20,30,31,32].

Clinical Implications

From a clinical perspective, the study underscores the importance of employing appropriate low-force systems for patients with compromised bone anchorage. Patients exhibiting reduced bone support are more susceptible to excessive tooth displacement and associated complications when subjected to conventional orthodontic forces. Thus, the study’s findings highlight the necessity for customized low-force application strategies for patients with diminished bone anchorage. Knowledge of these biomechanical characteristics is important for the development of effective orthodontic treatment methods aimed at mitigating the adverse effects of bone and root resorption to optimize treatment outcomes.

## 5. Conclusions

In conclusion, this study highlights the critical influence of bone resorption on initial tooth displacement during various orthodontic movements, with circumferential resorption posing the greatest challenge. The findings underscore the need for clinicians to consider both bone and root resorption when planning orthodontic treatments, particularly for patients with severe periodontitis, to mitigate the risk of excessive displacement and associated complications. Customizing low-force application strategies for patients with compromised bone support is essential to optimize orthodontic treatment outcomes and minimize the adverse effects of resorption. Addressing the study’s limitations in future research will further refine and enhance the clinical relevance of these findings.

## Figures and Tables

**Table 1:** Material parameters of the model structures utilized in the investigation.

Material	Young’s Modulus (MPa)	Poisson’s Ratio μ
Tooth (average value)	20,000	0.30
Bone (average value)	2000	0.30
Periodontal ligament	Bilinear, E1 = 0.05/E2 = 0.20 ε12: 7.0%	0.30

Material parameters of the model structures utilized in the investigation taken from Poppe et al., 2002 [18].

## Data Availability

Data are contained within the article.
